# Weakening of annual temperature cycle over the Tibetan Plateau since the 1870s

**DOI:** 10.1038/ncomms14008

**Published:** 2017-01-17

**Authors:** Jianping Duan, Jan Esper, Ulf Büntgen, Lun Li, Elena Xoplaki, Huan Zhang, Lily Wang, Yongjie Fang, Jürg Luterbacher

**Affiliations:** 1 State Key Laboratory of Vegetation and Environmental Change, Institute of Botany, Chinese Academy of Sciences, 100093 Beijing, China; 2 Department of Geography, Justus Liebig University of Giessen, 35390 Giessen, Germany; 3 Department of Geography, Johannes Gutenberg University, 55099 Mainz, Germany; 4 Swiss Federal Research Institute WSL, 8903 Birmensdorf, Switzerland; 5 Oeschger Centre for Climate Change Research, CH-3012 Bern, Switzerland; 6 Global Change Research Centre and Masaryk University, 61300 Brno, Czech Republic; 7 Chinese Academy of Meteorological Sciences, 100081 Beijing, China; 8 Institute of Geographic Sciences and Natural Resources Research, Chinese Academy of Sciences, 100101 Beijing, China; 9 National Climate Center, China Meteorological Administration, 100081 Beijing, China; 10 Centre of International Development and Environmental Research, Justus Liebig University of Giessen, 35390 Giessen, Germany

## Abstract

The annual cycle of extra-tropical surface air temperature is an important component of the Earth's climate system. Over the past decades, a reduced amplitude of this mode has been observed in some regions. Although attributed to anthropogenic forcing, it remains unclear when dampening of the annual cycle started. Here we use a residual series of tree-ring width and maximum latewood density from the Tibetan Plateau >4,000 m asl to reconstruct changes in temperature seasonality over the past three centuries. The new proxy evidence suggests that the onset of a decrease in summer-to-winter temperature difference over the Tibetan Plateau occurred in the 1870s. Our results imply that the influence of anthropogenic forcing on temperature seasonality might have started in the late nineteenth century, and that future human influence may further contribute to a weakening of the annual temperature cycle, with subsequent effects on ecosystem functioning and productivity.

## Abstract (web summary)

Weakening of the Tibetan Plateau's annual temperature cycle has been observed in recent decades, yet the long-term context remains unknown. Here, the authors reconstruct a 300-year temperature record from tree-ring width and maximum latewood density, which indicates the onset of weakening as early as the 1870s.

The annual temperature cycle is the dominant variability of many climate variables outside the tropics^1^,^2^,^3^,^4^. Changes in the amplitude of the annual temperature cycle not only affect the estimation of climate trends and variability (for example, the classification of El Niño/La Niña years^3^) but also have strong influences on biological and ecological systems (for example, plant developmental genetics^5^, bird and mammal distributions^6^ and insect population dynamics^7^). Superimposed on the rapid warming trend over the past decades, the annual temperature cycle weakened in several regions^3^,^8^,^9^. Moreover, observational and model-based studies suggest that recent anthropogenic forcing had a detectable influence on the annual cycle of surface air temperature^3^,^9^,^10^. However, due to the limited length of instrumental temperature records, it remains unclear when the weakening of the annual temperature cycle actually started and how recent behaviour compares to past natural climate variability.

The Tibetan Plateau (TP) is known as the third pole of the world, and responds more sensitively to climate change than other zones in the same latitude^11^,^12^,^13^. In the instrumental period, this region experienced a pronounced weakening of temperature seasonality (−0.18 °C per decade over the 1955–2011 period; Supplementary Figs 1 and 2). For the long-term understanding of temperature seasonality variability, proxy archive has become an essential necessity. The sensitivity of most biological proxy archives, including tree rings, is restricted to the warm season during which growth is triggered by fluctuations in ambient temperature and/or water availability. However, information on past winter conditions from tree rings only exists at a few sites^14^,^15^,^16^,^17^,^18^,^19^,^20^,^21^. Cell formation of high-elevation *Picea likiangensis var. balfouriana* trees in the southeastern part of the TP >4,000 m asl is one of these sites controlled by winter temperatures^18^,^19^,^20^, whereas cell wall thickening is primarily driven by late summer warmth^22^,^23^,^24^,^25^. Low winter temperatures have been demonstrated to effectively reduce radial growth rates by the interaction between snow cover and fine root mortality^21^,^26^,^27^,^28^,^29^. A deep snow pack in late winter has been shown to effectively reduce radial growth rates by maintaining low soil temperatures and delaying the initiation of cambial activity^21^,^29^. Nevertheless, further work on *Picea likiangensis var. balfouriana* tree growth responses to winter temperature is still needed to provide a more detailed mechanistic understanding.

Here we provide new evidences on the annual temperature cycle using tree-ring width (TRW) and maximum latewood density (MXD) data from the TP trees near the upper treeline >4,000 m asl. Our results show that the reduced temperature seasonality identified in instrumental data^3^ actually started in the 1870s over the TP. Before this time, a slight increasing trend in temperature seasonality is recorded, whereas persistent weakening is recorded thereafter. This trend can be confirmed using large-scale observational gridded data and agrees with model-simulated seasonality estimates. The onset of this shift coincides with the beginning of an anthropogenic-induced atmospheric sulfate concentration increase on the TP.

## Results

### Climatic signals in TRW and MXD

**Figure 1:**
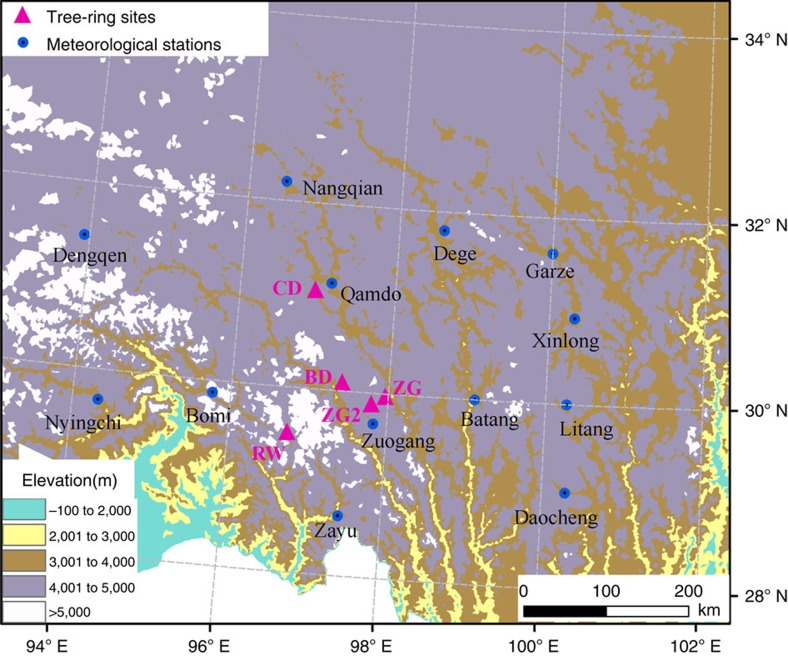
Map showing the locations of tree-ring sampling sites and meteorological stations on the Tibetan Plateau. Specific information of latitude, longitude and elevation for each tree-ring site and meteorological station is listed in Supplementary Tables 1 and 4. This map was created using the software ArcMap 9.2.

TRW and MXD data were obtained from 153 trees (175 cores) at five sites near the upper treeline in >4,000 m asl on the southeastern TP (Fig. 1). The longest series reaches back to 1509 and the shortest to 1688 (Supplementary Table 1; Supplementary Fig. 3). Significant correlations of the TRW and MXD site chronologies (Supplementary Tables 2 and 3) indicate that regional TRW and MXD composite records can be produced. Assessments of climate–growth relationships using Pearson correlation coefficients show that both the site and composite MXD chronologies contain strong summer temperature signals, while the TRW chronologies respond significantly to winter temperature (Supplementary Figs 4 and 5). The climatic signals are strongest in the regional composite chronologies, including a maximum response to November–February temperature in TRW and to July–September temperature in MXD. These seasonal patterns are consistent with previous studies^18^,^19^,^20^,^22^,^23^,^24^,^25^.

To examine the influence of potential co-linearities among climate variables on the climate–growth relationships, responses patterns are additionally assessed using the Seascorr program^30^ (see Methods), which revealed identical results (Supplementary Figs 6 and 7). Partial correlation coefficients show that MXD has a nonsignificant response to July–September precipitation and that TRW has a nonsignificant response to previous November to February precipitation. However, previous autumn and winter precipitation show significant partial correlations with the regional MXD and TRW, respectively. While there is not an obvious physiological explanation for the previous autumn precipitation signal in MXD, the significant partial correlation between TRW and previous winter precipitation might indicate an influence of the snow cover on the subsequent cambial activity^21^,^26^. Partial correlation analysis (see Methods) also shows that the correlations between MXD and winter temperature and between TRW and summer temperature are nonsignificant (grey bars in Supplementary Fig. 5). This indicates that there is no covariation influence of winter and summer temperature on the climate–growth relationships. For further analyses, we define summer temperature as the average of July to September and winter temperature as the average of the previous November to February.

### Tree-ring residual time series

To assess long-term changes in the annual temperature cycle, we establish three tree-ring residual time series (TMres) derived from the difference between MXD and TRW (that is, MXD minus TRW; see Methods). TMres1 is a time series derived from the detrended residual (MXD minus TRW) of a single series (for each core) and subsequent biweight averaging. TMres2 is a residual series (MXD minus TRW) obtained from the detrended MXD and TRW chronologies. TMres3 is a time series established by averaging all the single residual series (MXD minus TRW) and subsequent detrending. Based on the consideration of the expressed population signal (EPS)^31^>0.85 and the obviously increased s.d. before 1700 resulting from decreased sample size (Supplementary Fig. 8), three residual series between 1700 and 2011 are used for further analyses. Summer minus winter temperature residuals are calculated as the difference of July–September mean temperature minus the previous November–February mean temperature using instrumental station data (Supplementary Table 4). The tree-ring residual time series (TMres1–3, Supplementary Fig. 9) correlate significantly with observational temperature residuals, but TMres1 shows the strongest correlation (Supplementary Table 4). This outcome indicates that the method used to establish TMres1 eliminated more non-climatic noise, although the other approaches (TMres2 and TMres3) also revealed significant correlations (see Methods for detail). TMres1 is used for further temperature seasonality analysis.

### Response of TMres1 to seasonal temperature difference

**Figure 2:**
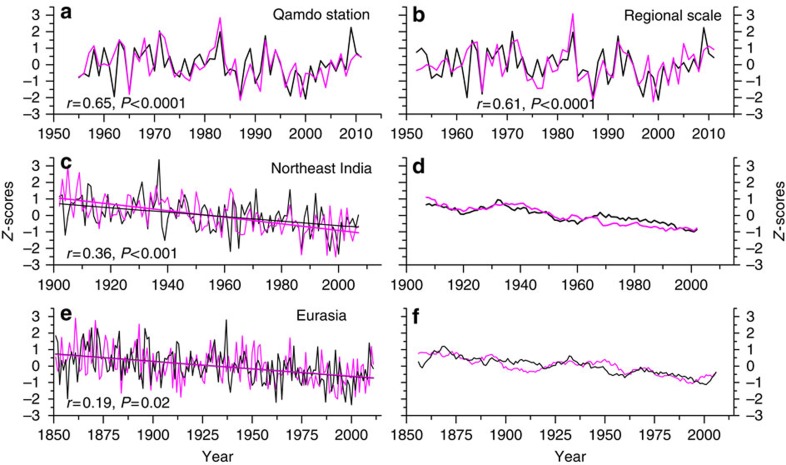
Comparison between TMres1 and instrumental temperature residual time series. (**a**) Comparison between the TMres1 and summer minus winter temperature difference in the Qamdo station over the 1955–2011 common period. (**b**) Comparison between the TMres1 and regional instrumental difference series (mean of 13 stations) over the 1952–2011 common period. (**c**) Comparison between the TMres1 and summer minus winter temperature difference in northeast India from 1902–2007. (**e**) Comparison between the TMres1 and the large-scale average (2.5–150° E, 25°–70° N) of gridded summer minus winter temperature time series from 1851 to 2011. (**d**,**f**) Eleven**-**year moving averages of the series shown in **c**,**e**. Lines in **c**,**e** indicate the linear trend.

When comparing TMres1 with the instrumental summer minus winter temperature difference, the highest correlations are recorded at the Qamdo and Xinglong stations (*r*=0.65; Supplementary Table 4). Since the Qamdo station covers a longer period from 1955 to 2011 (Xinglong from 1961 to 2011), this station, as well as a regional average, is used for further analysis (Fig. 2a,b). The results indicate that TMres1 is a good representative of interannual variability of the annual temperature cycle for the local and regional scales. Comparisons of TMres1 with the summer minus winter temperature difference recorded in northeastern India and the large-scale Eurasia mean (2.5–150° E, 25–70° N; Fig. 2c–f) reveal good agreements at the decadal scale and in the long-term trend. This outcome indicates that long-term changes in the annual temperature cycle as reconstructed for the TP are potentially representative for a larger region.

### Reconstruction of summer minus winter temperature difference

**Figure 3:**
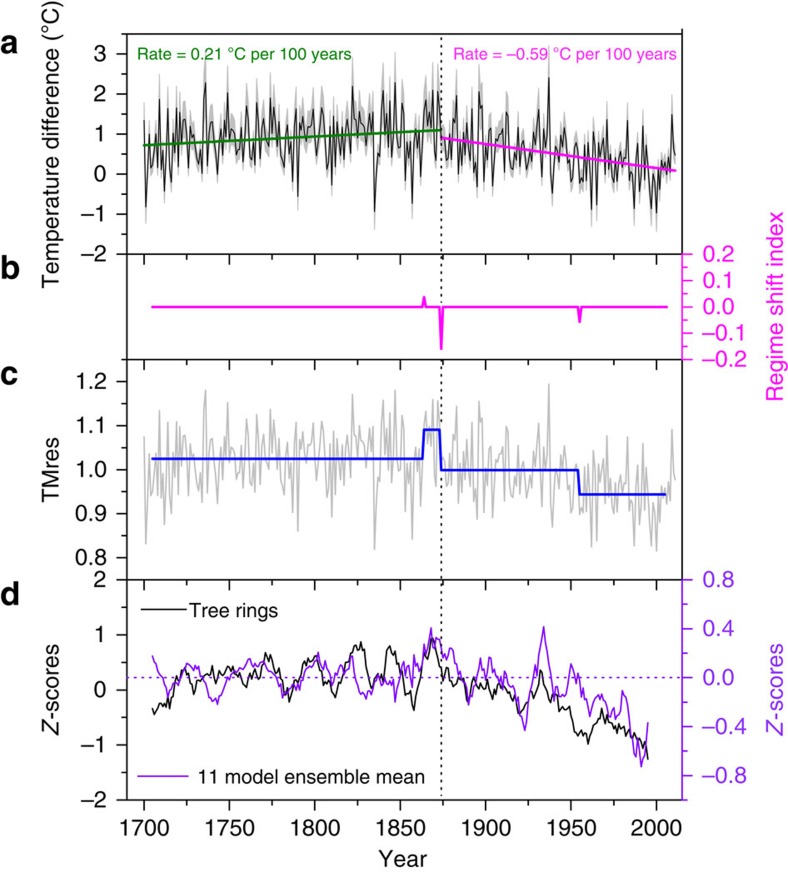
Temperature seasonality changes reconstructed from TP tree rings and its comparison with the multi-model ensemble mean. (**a**) Linear regressions fitted to the reconstructed temperature seasonality changes (black) over the 1700–1873 (olive) and 1874–2011 (magenta) periods. (**b**) Regime shift index for the regime shift detection in the TMres1 (arrows indicate the turning points and theirs shift direction, up means increase and down means decrease). (**c**) Mean changes of the regime shift detection in the TMres1 series. (**d**) Comparisons between normalized TMres1 and summer-versus-winter temperature residuals derived from an ensemble mean of 11 models over the 1700–2000 period (Supplementary Table 6). Note that curves in **d** are the 11-year moving average. The grey shaded area in **a** indicates the 95% confidence interval of the reconstruction series (black).

To capture the strongest signal of temperature seasonality on the TP, the summer–winter residual recorded at the Qamdo station was chosen as the reconstruction target. The highly significant correlation (*r*=0.81, *n*=59, *P*<0.0001) between the Qamdo and regional residual series indicates the representativeness of the station record for larger spatial scales. The skill of the transfer regression model is verified using leave-one-out cross-validation methods (Supplementary Table 5)^32^. The reconstructed seasonal temperature difference back to 1700 shows a change in trend behaviour over time (Fig. 3a), and regime shift detection^33^ reveals three turning points in 1863, 1874 and 1955 (Fig. 3b,c). The year 1863 indicates the starting point of an increasing trend, while 1874 and 1955 mark the start of trend decreases. Overall, a slightly increasing trend is recorded before the 1870s (0.21 °C per 100 years from 1700 to 1873) and is replaced by an almost threefold negative trend thereafter (−0.59 °C per 100 years). The same trend changes are revealed in the model-simulated temperature seasonality estimates of the TP (Fig. 3d).

**Figure 4:**
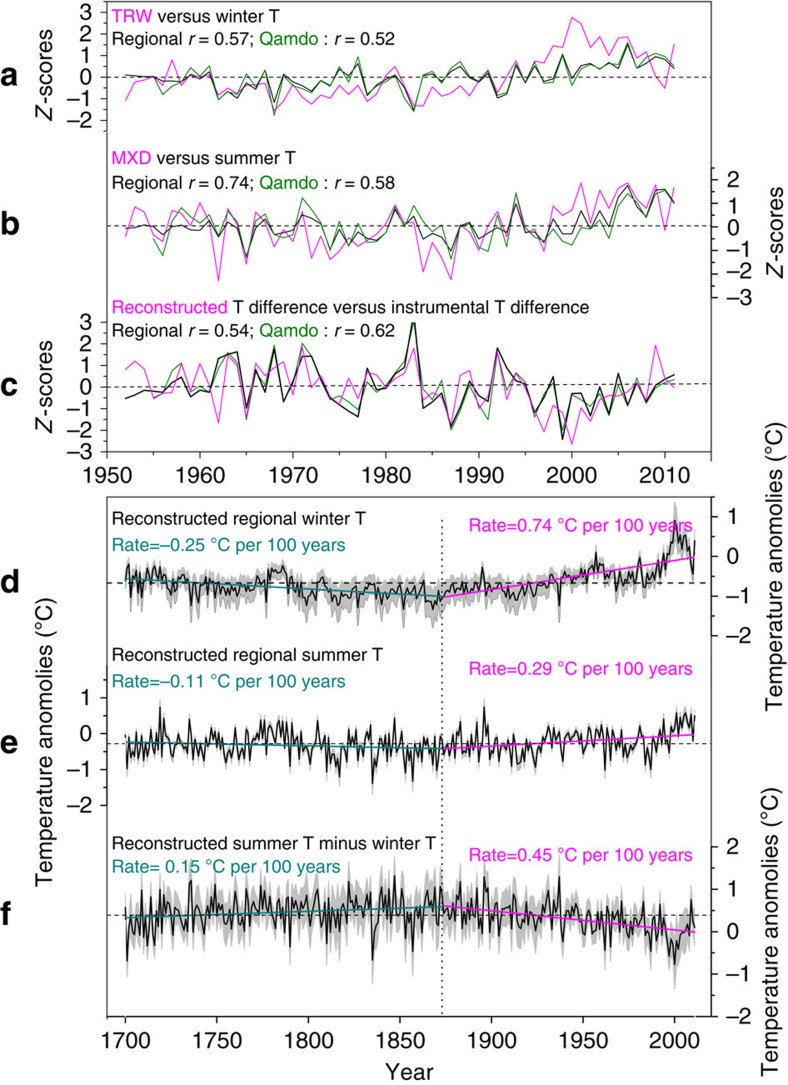
TRW- and MXD-based winter and summer temperature reconstructions. (**a**) Comparisons of the regional TRW chronology (magenta) with winter temperature (previous November to February) records in regional average (black) and the Qamdo station (olive). (**b**) Comparisons of the regional MXD chronology (magenta) with summer temperature (July–September) records in regional average (black) and the Qamdo station (olive). (**c**) Comparisons of the reconstructed summer temperature minus reconstructed winter temperature (magenta) with instrumental summer temperature minus instrumental winter temperature in regional average (black) and the Qamdo station (olive). (**d**) Winter temperature reconstruction derived from the regional TRW chronology (anomalies wrt 1981–2010). (**e**) Summer temperature reconstruction derived from the regional MXD chronology (anomalies wrt 1981–2010). (**f**) The difference between summer temperature reconstruction and winter temperature reconstruction over the period 1700–2011. Grey shaded areas in **d**–**f** indicate the 95% confidence interval of the reconstruction series (black).

To further assess seasonal temperature change, we performed independent summer and winter temperature reconstructions using MXD and TRW, respectively (Fig. 4; Supplementary Table 5). Reconstructed regional winter and summer temperatures explain 32.5% and 54.8% of the variance in the instrumental records, respectively (Fig. 4d,e). Although the MXD and TRW chronologies show higher correlations with regionally averaged temperature than with the Qamdo station record (Fig. 4a,b), the summer minus winter temperature differences derived from the summer and winter temperature reconstructions have a better match with summer minus winter temperature differences recorded at the Qamdo station (*r*=0.62) compared with regional averages (*r*=0.54; Fig. 4c). This finding is similar to the result obtained from TMres1 (Fig. 2a,b), but TMres1 has a higher correlation with the record at the Qamdo station (*r*=0.65; Fig. 2a,b). Additional analyses show that the variance of summer minus winter temperature difference explained by independent summer and winter temperature reconstructions has more similarity to that explained by the Qamdo station records than by the regional average (Methods). This similarity is the likely reason for why the reconstructed summer minus winter temperature series correlates much higher with records from the Qamdo station than the regional average.

Both the winter and summer temperature reconstructions show a smaller trend before the 1870s than thereafter. Since the 1870s, a larger increase rate can be found in the winter temperature reconstruction compared with the summer temperature reconstruction (0.74 °C per 100 years for winter and 0.29 °C per 100 years for summer; Fig. 4d,e). In contrast to the summer minus winter temperature differences derived from TMres1 (Fig. 3a), the result obtained from the winter and summer temperature reconstructions show a smaller downward trend since the 1870s (Fig. 4f). This trend likely occurred because the latter reconstructions have a larger uncertainty and less variance explained of instrumental data (Fig. 4). Therefore, both the summer minus winter temperature differences derived from the two methods demonstrate the persistent weakening of the annual temperature cycle since the 1870s, but TMres1 as the proxy is a better choice.

### Spatial characteristics of the annual temperature cycle

**Figure 5:**
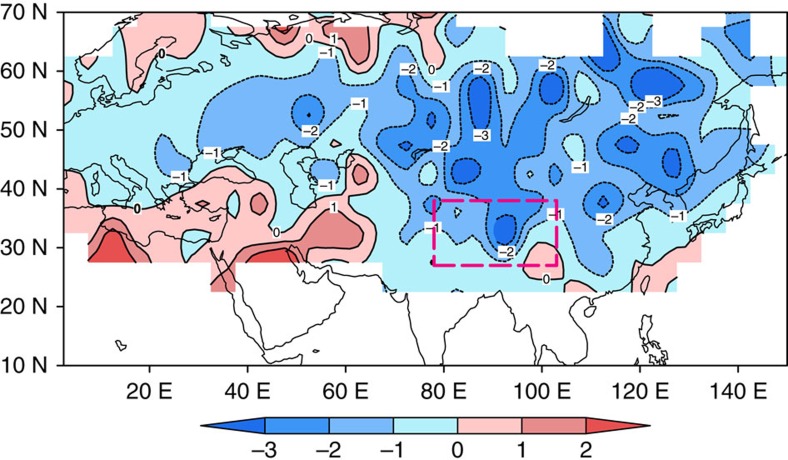
Large-scale trend of summer minus winter temperature differences from 1874 to 2011. The trend (°C per 100 years) was calculated using the CRU grid temperature data set (CRUTEM.4.3). The pink rectangle denotes the Tibetan Plateau. For data availability of each grid, please see Supplementary Fig. 11.

To assess the spatial characteristics of annual temperature cycle and to verify our proxy-based reconstruction against observational data, both extreme years and trends identified in the reconstruction are analysed using instrumental and model-simulated temperatures (Supplementary Table 6). Two extremely positive years (1983 and 1992) and two extremely negative years (1987 and 1999) show consistent spatial variations across the TP (Supplementary Fig. 10). The distance-related correlation decay of TMres1 with observed summer minus winter temperature differences (Fig. 2a,c,e) indicates that interannual variability of the annual temperature cycle differs between the TP and the remote regions. However, the consistent weakening trend (Figs 2d,f and 5) demonstrates that a long-term weakening of temperature seasonality since the 1870s occurred at larger spatial scales. The trend of the annual cycle shown in Fig. 5 in regions with short instrumental data (that is, 1950s–2011; Supplementary Fig. 11) is identical with the observed change on the TP (about −2 °C per 100 years; Supplementary Fig. 1). Regions with a longer observational data (that is, 1874–2011; Supplementary Fig. 11) reveal trends close to our reconstructed change rate (near −1 °C per 100 years; Fig. 3a).

The comparison of TMres1 with seasonality estimates derived from an ensemble of 11 global climate models (Supplementary Table 6) provides additional evidence of reduced temperature seasonality. Although the interannual consistence is weak and there is also partial disagreement in decadal variability (for example, 1740s–1750s and 1700s–1710s), the obvious turning point in temperature seasonality in the 1870s can be seen in both the proxy- and model-based evidence (Fig. 3c,d).

### Possible drivers of the weakened temperature seasonality

Studies based on observational data from the TP showed that cold season temperatures experienced a greater warming rate than warm season temperatures over the last decades^34^. Similar results were obtained in other regions of the Northern Hemisphere land mass^3^. For the different seasonal warming rates in high northern latitudes, East Asia and Europe, model-simulated studies indicate that anthropogenic forcing is the most important driver^3^,^35^. On longer timescales, ice-core records reveal that atmospheric sulfate concentrations on the TP were relatively low and constant in the period 1000–1870, but thereafter, concentrations increased significantly^36^. The main sources of the increased sulfate concentrations since 1870 on the TP are almost certainly anthropogenic in origin^36^. Sulfate aerosols force climate by reflecting sunlight into space and also by acting as condensation nuclei, which tend to make clouds more reflective and change their lifetimes, causing a net cooling^37^,^38^. It has been suggested that atmospheric sulfate has a greater insolation in summer than winter and that the shortwave radiative influence of anthropogenic sulfate aerosol is greater in summer leading to a decreasing warming rate as compared with winter^39^,^40^. These results imply that the human-induced increase of atmospheric sulfate concentrations might be responsible for the persistent weakening of temperature seasonality over the TP since the 1870s.

In this study, a novel approach of using MXD minus TRW residuals shows a better ability in capturing seasonal temperature signals compared with the traditional method (that is, using TRW and MXD chronologies; see Methods). This new approach can be used to extract information on climate seasonality in regions where TRW and MXD are distinctively sensitive to climatic signals in different seasons. Importantly, our study indicates an early shift of changing temperature seasonality that already started in the late 19th century on the TP, and it was likely associated to changes in atmospheric sulfate concentrations. These results imply that future alterations of atmospheric composition driven by human activity might further influence the temperature seasonality, triggering phenology changes (for example, earlier phase shift^1^) and ecological effects (for example, plant developmental genetics^5^ and animal distributions^6^,^7^).

## Methods

### Tree-ring data and residual series

Tree-ring density of old growth Balfour spruce (*Picea likiangensis var. balfouriana*) was measured using the X-ray method according to standard practices^41^. Both the TRW and MXD data were derived from the resultant X-ray image, and the measured tree-ring sequences were absolutely cross-dated by matching the ring width/density patterns among the trees. In total, 175 core samples from five sites were successfully cross-dated (Supplementary Table 1).

Three tree-ring-based residual time series (TMres1, TMres2 and TMres3) were developed to assess long-term changes in the annual temperature cycle. TMres1 is established as follows. First, each MXD and TRW series was normalized to the zero mean and unit s.d. Second, a residual time series (MXD minus TRW) was calculated for each core sample. Third, the single core residual time series were detrended using the ‘Signal-Free' technique with RCSsigFree software^42^, in which a linear fit and subsequent age-dependent spline standardization were applied to the individual series. A convergence test was set to 0.0005 minimizing the mean absolute difference between the current and the preceding series, with the limit reached at the second iterative step. Fourth, individual residuals series (that is, MXD minus TRW) were calculated as the ratio between the actual and modelled values, and regional residual series were calculated using the biweight robust mean of all single residual series (Supplementary Fig. 9a). Finally, the reliable period of the regional residual series (1700–2011) was determined considering an EPS >0.85 as well as s.d. changes before 1700 resulting from decreased sample sizes (Supplementary Fig. 8c,d).

TMres2 is the difference of the regional MXD chronology minus the regional TRW chronology. It is established using the following three steps. First, calculate the regional MXD and TRW chronologies based on all tree-ring series of the five sampling sites using the Signal-Free approach. For the detrending, a linear fit was applied to MXD, and age-independent spline standardization was applied to TRW. Chronology indices were calculated as ratios, and the convergence criterion was set to 0.005. Second, the detrended MXD and TRW chronologies were normalized to the zero mean and unit s.d. from 1700 to 2011. Finally, a residual time series between the normalized MXD and TRW chronologies was calculated over the period 1700–2011.

TMres3 is also a residual series of MXD minus TRW. It is established based on the following steps. First, each MXD and TRW series was normalized to the zero mean and unit s.d. Second, a residual time series (MXD minus TRW) was calculated for each core sample. Third, all the single core residual time series were averaged to establish a regional seasonality estimate. Fourth, the regional residual time series was detrended using the Signal-Free approach, in which a linear fitting and subsequent age-dependent spline standardization was used. The convergence test was set to 0.0005, and the residuals (that is, MXD minus TRW) were derived as the ratio between the actual and modelled values. Finally, the reliable period of the regional residual series (1700–2011) was determined considering an EPS >0.85 as well as standard deviation changes prior to 1700 resulting from decreased sample sizes (Supplementary Fig. 8c,d).

TMres1 and TMres2 reach a correlation of 0.97 over the period 1700–2011, TMres1 and TMres3 correlate at 0.96, and TMres2 and TMres3 at 0.94, all reaching *P*<0.0001 (Supplementary Fig. 9). The correlation coefficients between the proxy-based residual time series and the summer minus winter instrumental temperatures show that TMres1 captures the seasonal temperature variations better than TMres2 and 3 and that TMres1 calibrates best (Supplementary Table 4).

To test the sensitivity of TMres1 to different detrending methods, we additionally performed the detrending of TMres1 using Arstan software^43^ with a negative exponential curve fit method (variance stabilization was used). The two resulting series derived from different detrending methods show a highly significant correlation (*r*_1700–2011_=0.98) and a consistent weakening trend since the 1870s (Supplementary Fig. 12). However, the TMres1 derived from the Signal-Free method reserve more medium-frequency variance^44^ (Supplementary Fig. 12) and show a better correlation with the instrumental summer minus winter temperature difference (*r* =0.65) than with the tree-ring residual series derived from the Arstan method (*r* =0.63).

For the detrending of regional and individual TRW and MXD site chronologies (Fig. 4; Supplementary Fig. 3), age-dependent spline and linear fit were used, respectively. All the indices of these chronologies were calculated as ratios, and the convergence criterion was 0.005.

### Climatic data

Monthly climate data from 13 meteorological stations around the five sampling sites are used in this study (Supplementary Table 4). The record length varies among the stations but includes the least 33 years from 1979 to 2011. The gridded data of land surface air temperature used in this study are from CRUTEM4.3 at a spatial resolution of 5° by 5° starting in 1850 (ref. 45). The homogeneous monthly surface temperature data in northeastern India was obtained from the Indian Institute of Tropical Meteorology (http://www.tropmet.res.in/static_page.php?page_id=54). This data set covers the period 1901–2007. For the specific spatial domain (that is, northeastern India) of this data set, please see the website http://www.tropmet.res.in/static_page.php?page_id=54. Model-simulated monthly temperature data covering the past millennium (past 1,000 experiment) from the CMIP5 archive (https://pcmdi.llnl.gov/search/cmip5/) is compared with the annual temperature cycle trend reconstructed using tree-ring data (Supplementary Table 6).

### Climate–growth analysis

Climate–growth relationships were first identified using Pearson correlation analysis (Supplementary Figs 4 and 5). Further examination of climate–growth relationships was also performed using the Seascorr program^30^ (Supplementary Figs 6 and 7). In these analyses, monthly mean temperature served as the primary climate variable to perform correlation analysis, while monthly total precipitation served as the secondary climate variable to perform partial correlation analysis. Correlation and partial coefficients are calculated both for individual months from August of the previous year through September of the growth year and for multi-months combined seasonal groupings (that is, 3, 4 and 6 months). The Seascorr program was also used to identify if winter temperatures (that is, November–February temperature) tend to covary with temperatures during the summer months (that is, July–September), influencing the climate–growth relationships. In these analyses, summer/winter temperature served as the primary climate variable to perform correlation analysis with MXD/TRW, while winter/summer temperature served as the secondary climate variable to perform partial correlation analysis with MXD/TRW. The calculation results of correlation coefficients are identical to Pearson correlation analysis (Supplementary Fig. 5), while the calculated partial correlation coefficients are marked as grey bars in Supplementary Fig. 5.

### Climate reconstructions

Linear regression^46^ was used to reconstruct the long-term changes in summer minus winter temperature difference (Fig. 3a), regional winter temperature (Fig. 4d) and regional summer temperature (Fig. 4e). In these reconstructions, the TMres1, regional TRW chronology and regional MXD served as independent variables while the summer minus winter temperature difference recorded in the Qamdo station and regional previous November–February mean temperature and regional July–September mean temperature severed as dependent variables. The skill of the regression models is verified using the leave-one-out cross-validation method^32^. All evaluation statistics reach a significance level of 0.01, indicating some statistical skill in the regression model (Supplementary Table 5).

### Variance explanations and uncertainties

Instrumental winter and summer temperature anomalies in the Qamdo station explain 46.2% and 29.2% of the variance in the summer minus winter temperature difference over the period 1955–2011, respectively. The regionally averaged winter and summer temperature anomalies explain 54.8% and 9.0% of the variance in the regional summer minus winter temperature difference over the period 1952–2011, respectively. The reconstructed winter and summer temperature anomalies (Fig. 4d,e) explain 24.0% and 31.4% of the variance in the difference of summer minus winter temperature anomalies (Fig. 4f) over the period 1700–2011, respectively. Thus, the variance of summer minus winter temperature difference explained by independent summer and winter temperature reconstructions has more similarity to that explained by the record in the Qamdo station than that explained by the regional average.

There are some differences between the temperature seasonality estimates derived from the TMres1 and from independent summer and winter temperature reconstructions. The former only experienced one detrending (that of TMres1) and is a direct reconstruction. However, the latter experienced two detrendings (those of TRW and MXD) and is an indirect difference of two reconstructed series. Thus, the temperature seasonality estimate derived from the difference of summer and winter temperature reconstructions could induce larger uncertainty (Fig. 4f, the uncertainty of summer temperature reconstruction plus the uncertainty of winter temperature reconstruction) than the TMres1-based direct reconstruction.

### Regime shift detection analysis

We performed regime shift detection analysis based on mean changes of the TMres1. For this analysis, the cutoff regime length was set as 12 and *P*=0.1. For detailed methods for the mean changes detection and the calculation of shift index please refer to ref. 33.

### Preprocessing of the model-simulated data

Simulations of surface monthly temperature from an ensemble of 11 models are used in the analysis. Supplementary Table 6 shows the number of simulations used from each model. Monthly anomalies of surface temperature are calculated for each grid point and simulations based on the reference period 1801–1830. The anomalies are then regridded to the common GISS-E2-R grid (2.5° × 2°) and masked to the TP range (27–38° N, 78–103° E). The ensemble mean of 11 models is obtained by first computing individual model ensemble means and then averaging their *Z*-scores across available models. The calculation gives equal weights for different models and thus avoids models with larger numbers of ensemble members dominating the statistics of the multimodal mean. Finally, the ensemble mean of 11 models in the period 1700–2000 is used for further analysis (Supplementary Table 6).

### Data availability

The data that support the findings of this study are available from the corresponding author upon request.

## Additional information

**How to cite this article:** Duan, J. *et al*. Weakening of annual temperature cycle over the Tibetan Plateau since the 1870s. *Nat. Commun.* **8,** 14008 doi: 10.1038/ncomms14008 (2017).

**Publisher's note**: Springer Nature remains neutral with regard to jurisdictional claims in published maps and institutional affiliations.

## Supplementary Material

Supplementary Information Supplementary Figures, Supplementary Tables and Supplementary References
