# Supplementary material for: Weakening of annual temperature cycle over the Tibetan Plateau since the 1870s
Source: Nat Commun. 2017 Jan 17;8:14008. doi: 10.1038/ncomms14008 (PMC5247579; doi:10.1038/ncomms14008)
Supplement: Supplementary Information — Supplementary Figures, Supplementary Tables and Supplementary References [file ncomms14008-s1.pdf]

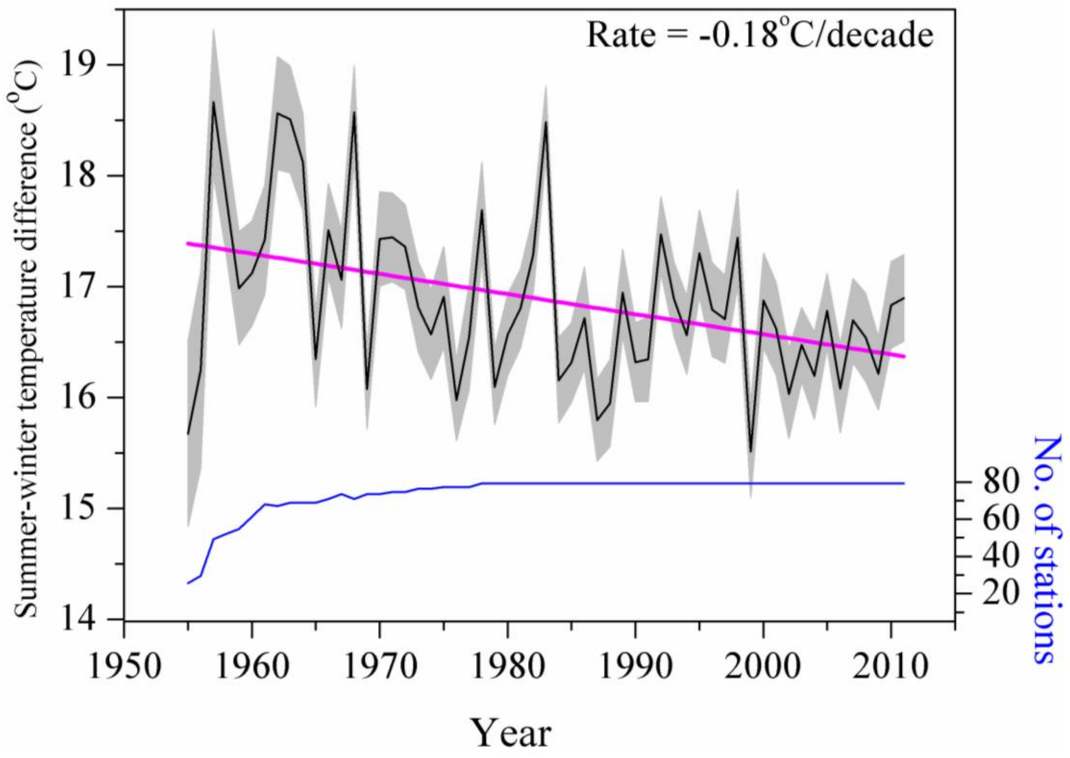

Supplementary Figure 1. Observational weakening of temperature seasonality over the Tibetan Plateau (TP). Note that the calculation of the temperature seasonality (i.e., July-September mean temperature minus November-February mean temperature) was based on data of 79 meteorological stations over the TP. The shaded area indicates one standard error. For the locations of the 79 meteorological stations please see the Supplementary Figure 2.

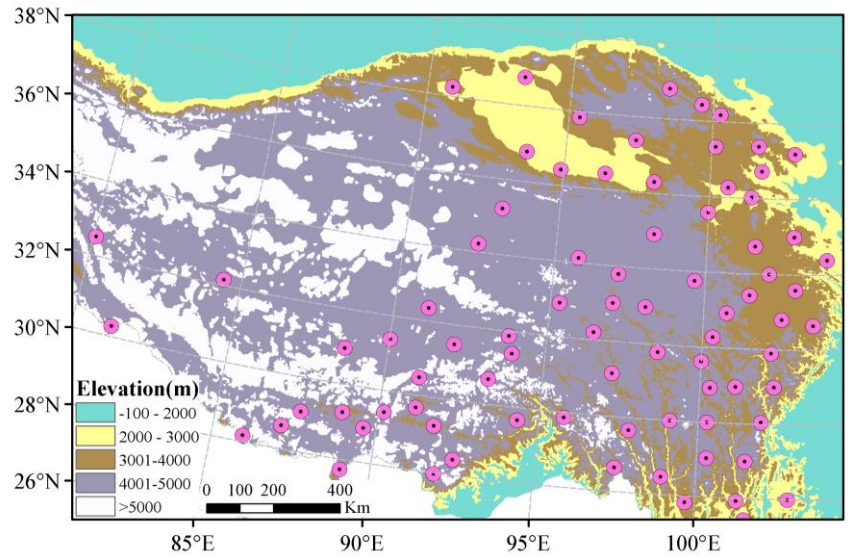

Supplementary Figure 2. Locations of the 79 meteorological stations used in the calculation of the temperature seasonality in Supplementary Figure 1. This map was created using the software ArcMap 9.2.

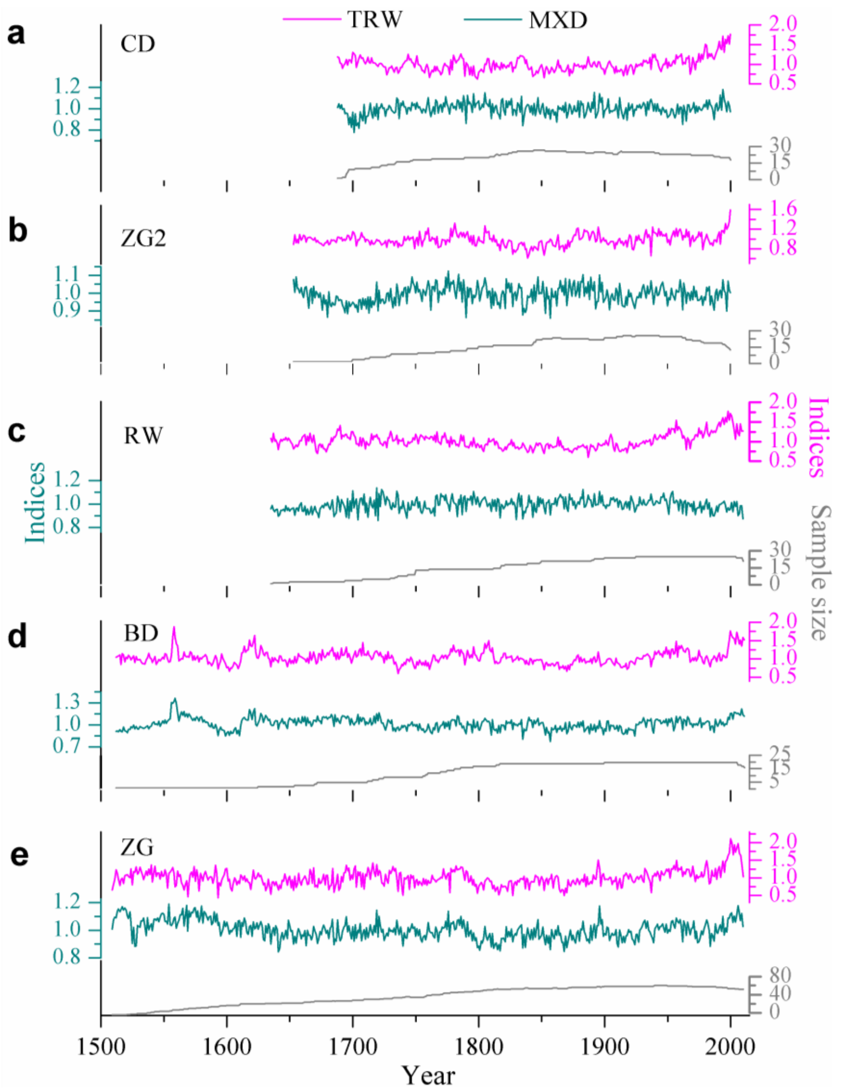

Supplementary Figure 3. Individual TRW and MXD site chronologies with sample size. (**a**) CD site. (**b**) ZG2 site. (**c**) RW site. (**d**) BD site. (**e**) ZG site.

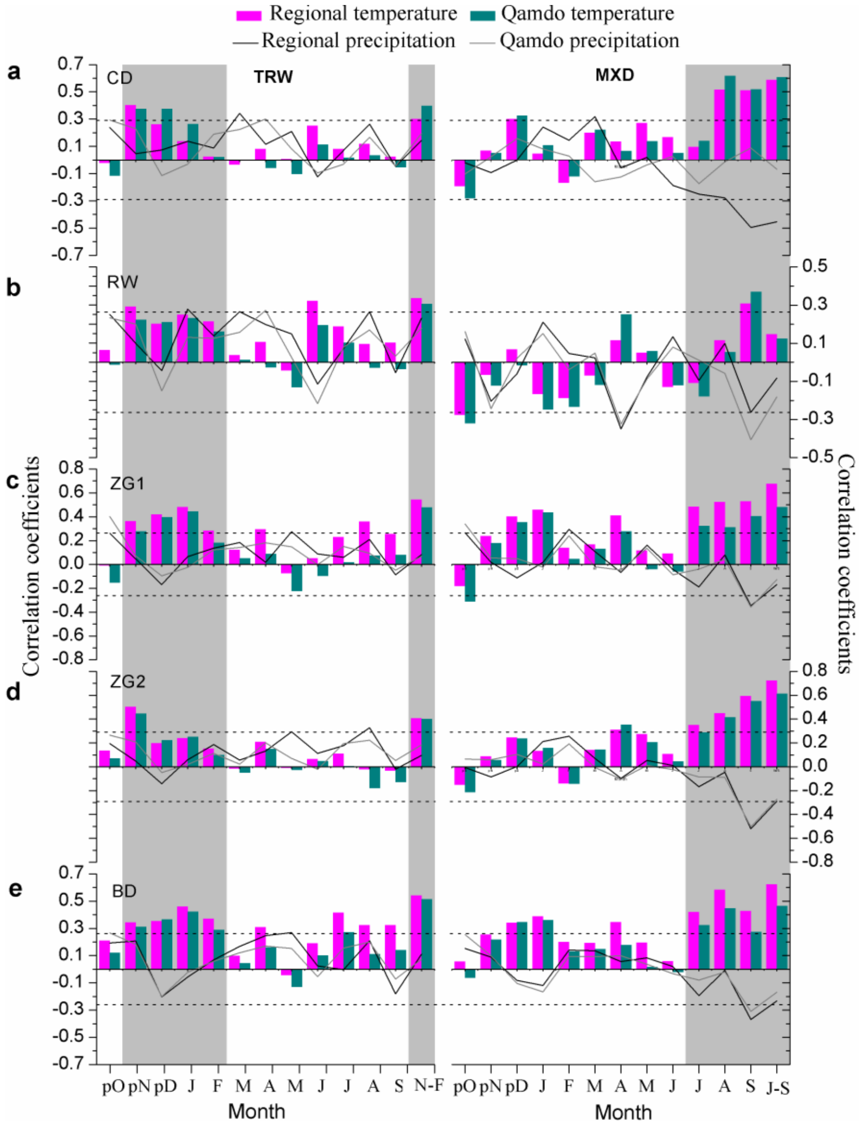

Supplementary Figure 4. Climate-growth relationships of individual TRW and MXD site chronologies with monthly temperature and precipitation. (**a**) CD site. (**b**) RW site. (**c**) ZG1 site. (**d**) ZG2 site. (**e**) BD site. Left and right columns are the correlation coefficients of individual TRW and MXD site chronologies with climate, respectively. The three grey shaded areas highlight highly significant correlation. “pO”, “pN” and “pD” mean previous October, November and December, respectively. J-S means July-September, N-F means previous November to February. The horizontal dotted lines indicate a statistical significance level of 0.01.

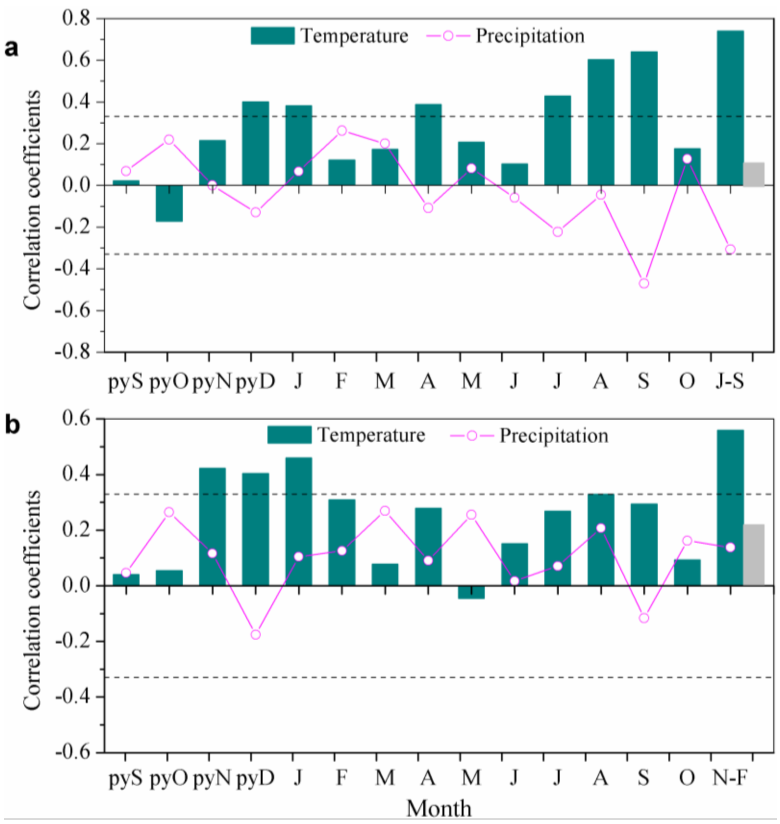

Supplementary Figure 5. Climate-growth relationships of the regional TRW and MXD chronologies with climate. (**a**) Correlation coefficient between the detrended regional MXD chronology and regionally monthly mean temperature and monthly precipitation. (**b**) Correlation coefficient between the detrended regional TRW chronology and regionally monthly mean temperature and monthly precipitation. "py" means previous year, J-S means July-September, N-F means previous November to February, horizontal dotted lines indicate a statistical significance level of 0.01. The grey bar in **a** is the partial correlation coefficients of the MXD chronology with winter temperature (i.e., previous November-February mean temperature), and in **b** is the partial correlation coefficients of the TRW chronology with summer temperature (i.e., July-September mean temperature).

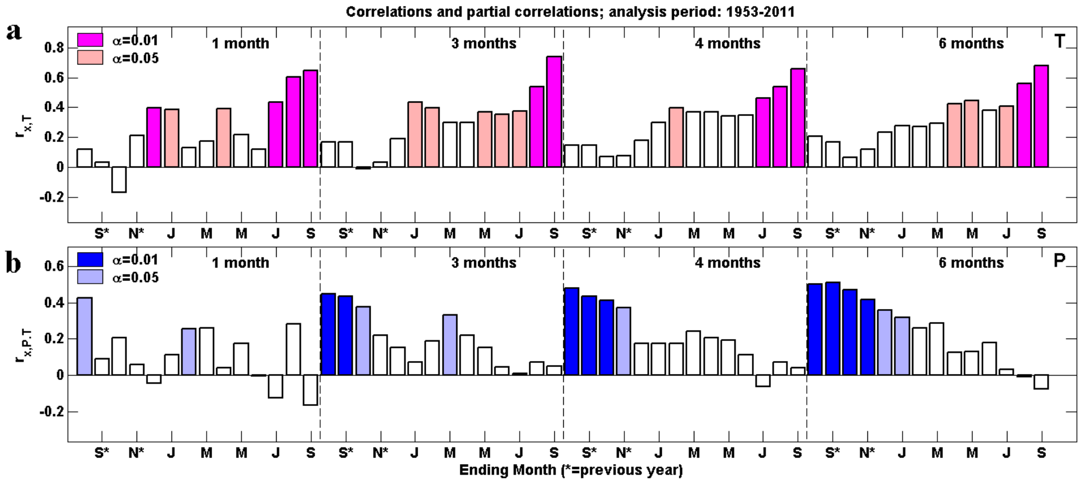

Supplementary Figure 6. Correlations and partial correlations of the regional MXD chronology with seasonality climate variables. (**a**) Correlations of the regional MXD chronology with the primary climate variable (monthly mean temperature, T). (**b**) Partial correlations of the regional MXD chronology with secondary climate variable (monthly total precipitation, P).

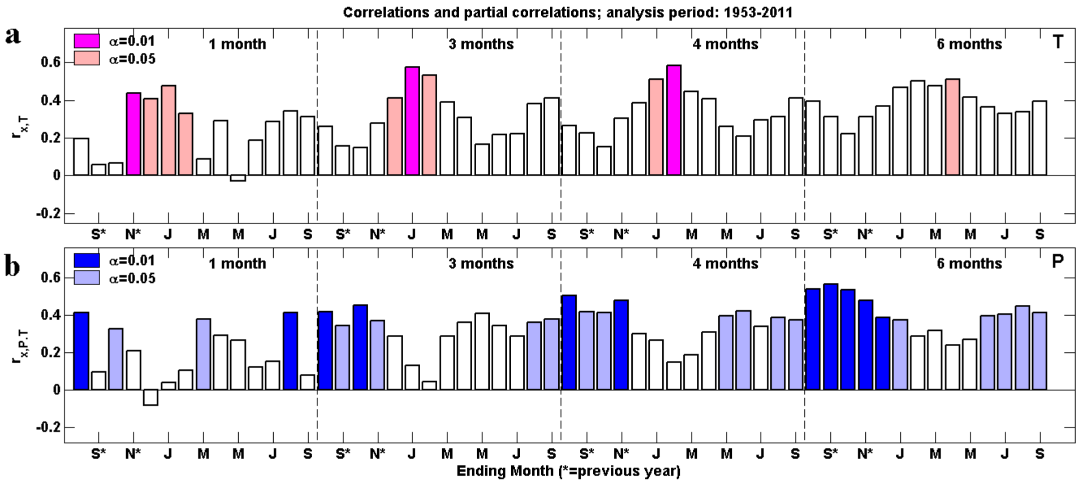

Supplementary Figure 7. Correlations and partial correlations of the regional TRW chronology with seasonality climate variables. (**a**) Correlations of the regional TRW chronology with the primary climate variable (monthly mean temperature, T). (**b**) Partial correlations of the regional TRW chronology with the secondary climate variable (monthly total precipitation, P).

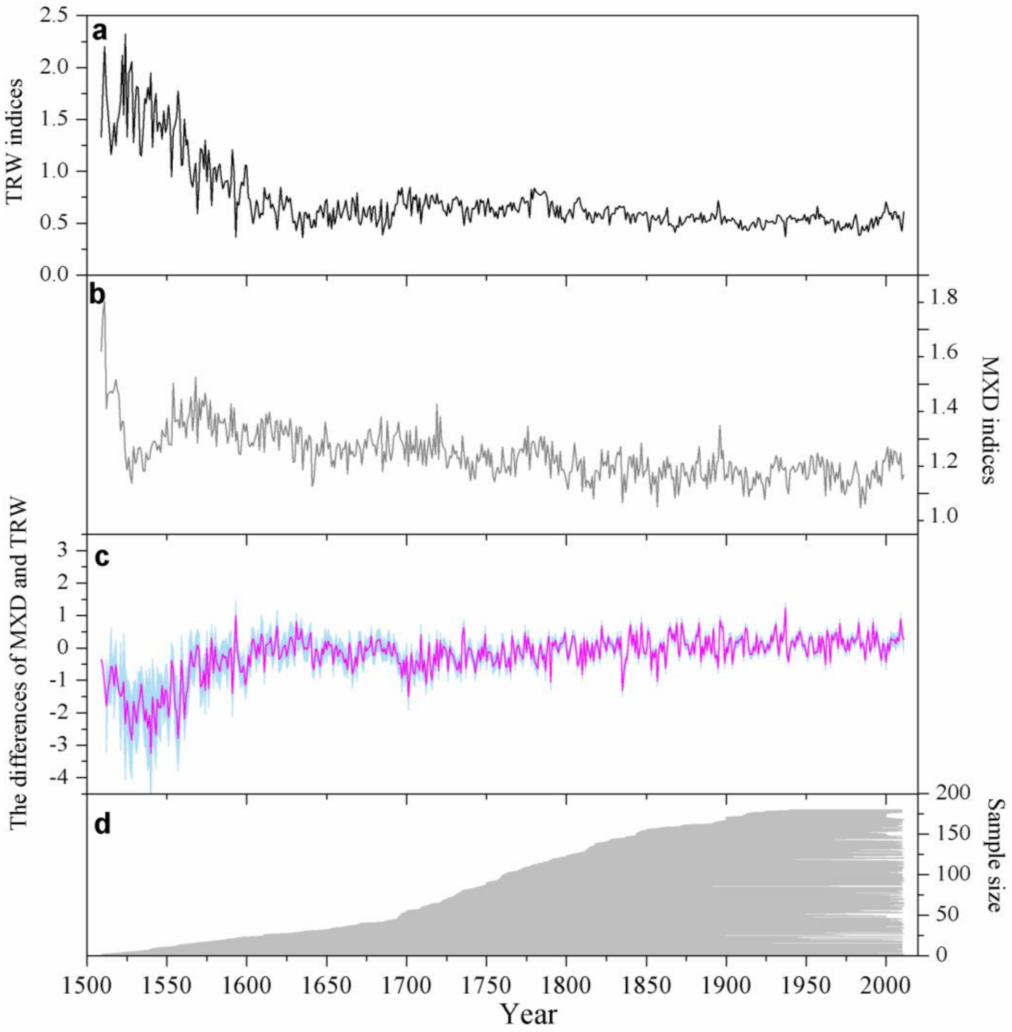

Supplementary Figure 8. Regional tree-ring raw chronology and tree-ring residual series (MXD minus TRW). (**a**,**b**) Regional TRW and MXD raw chronologies established using the tree-ring series from 5 sampling sites (Fig. 1). (**c**) Regional averaged tree-ring residual series (normalized MXD minus normalized TRW) of all tree-ring series from the 5 sampling sites (Fig. 1). Blue shading in **c** denotes 2 standard errors from the mean. (**d)** The sample depth. All tree samples are ordered by the calendar age of their innermost ring.

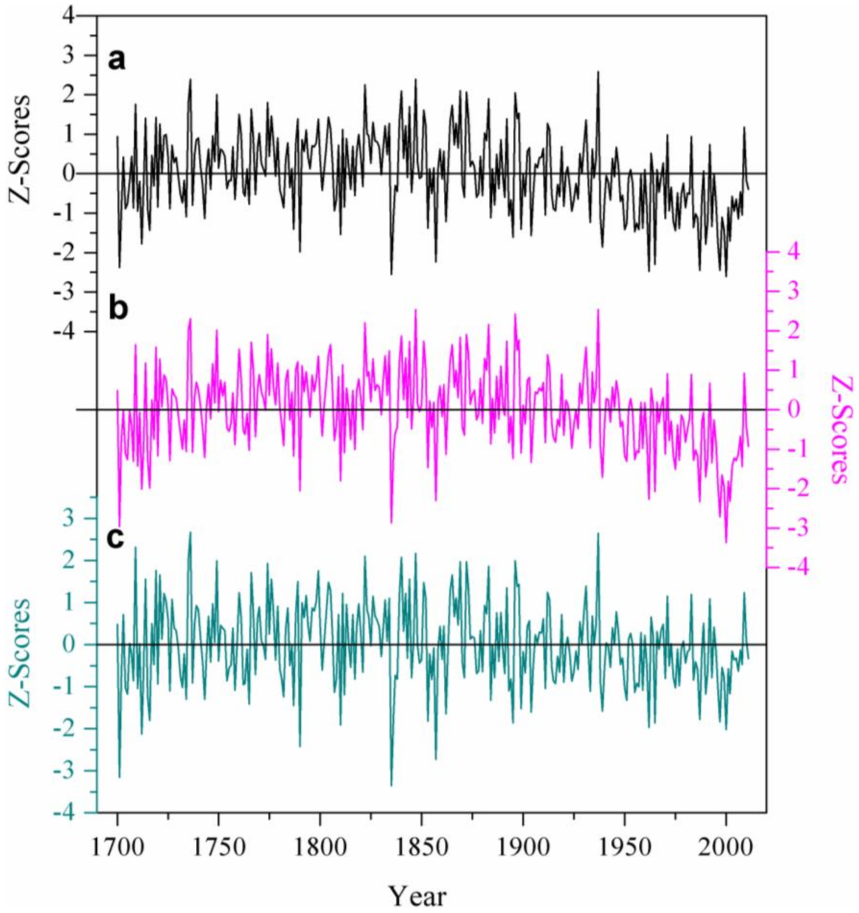

Supplementary Figure 9. Detrended regional tree-ring residual series over the period 1700-2011. (**a**) TMres1. (**b**) TMres2. (**c**) TMres3. The methods used to establish three residual series are described in the Methods.

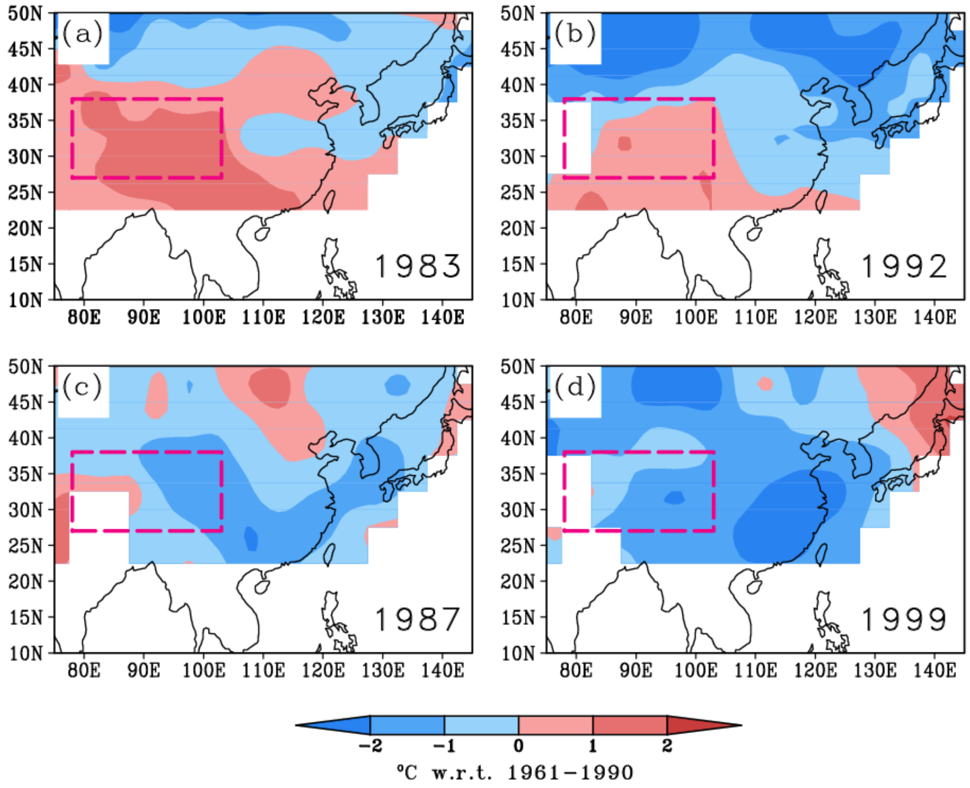

Supplementary Figure 10. Spatial characteristics of extreme years of summer-winter temperature residuals during the instrumental period (1952-2011). (**a**,**b**) The 1983 and 1992 positive summer-winter temperature deviations. (**c**,**d**) The 1987 and 1999 negative temperature seasonality deviations. The rectangles denote the Tibetan Plateau. This figure was created using the software GrADS 1.9.

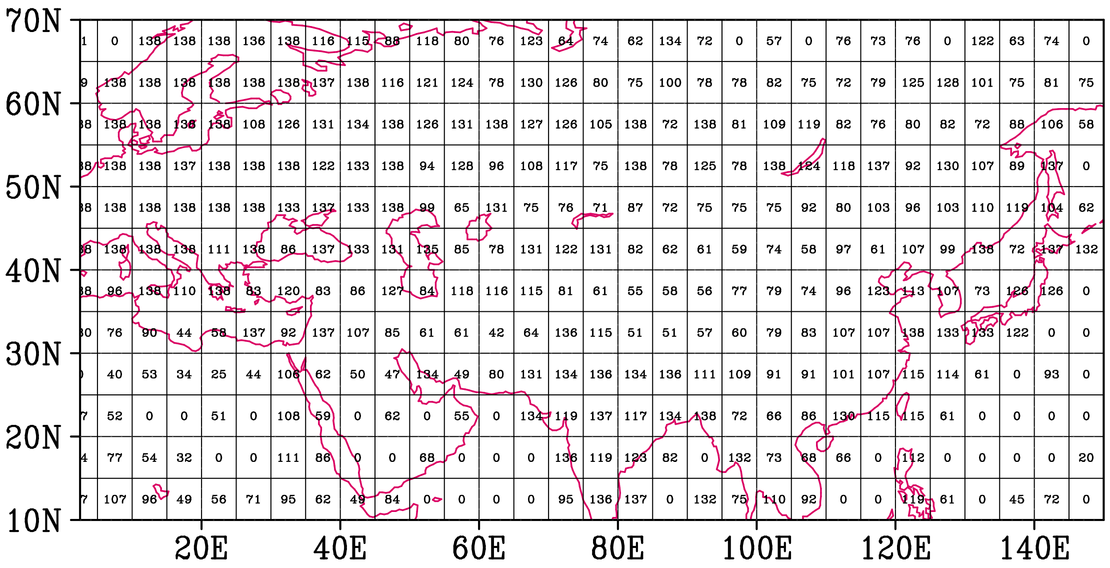

Supplementary Figure 11. Data availability (i.e., number of years with data) for calculation of the change rate of summer-winter temperature residuals in the period 1874-2011 for each grid in Fig. 5. This figure was created using the software GrADS 1.9.

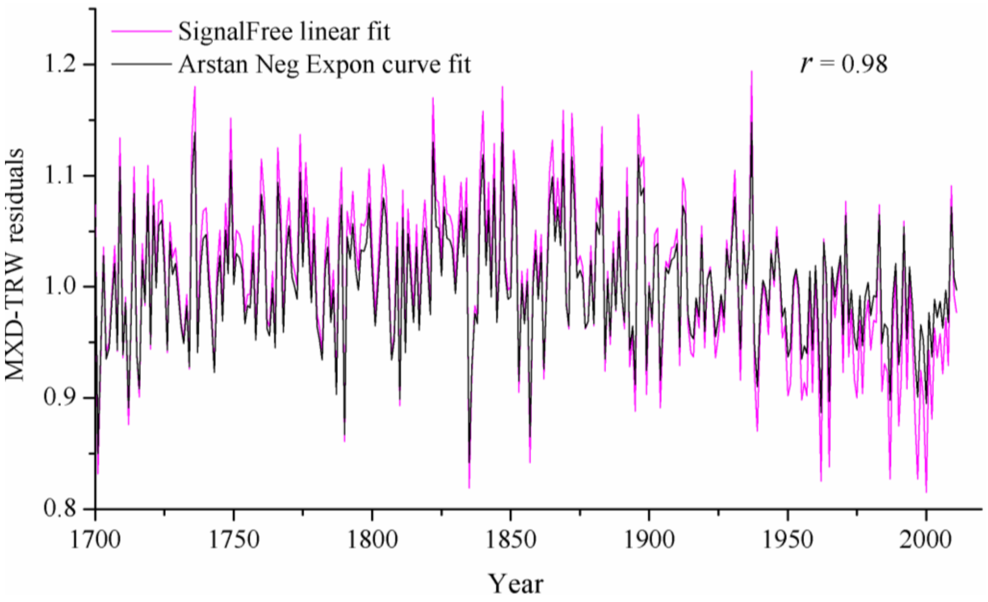

Supplementary Figure 12. Comparison of the tree-ring residual series derived from different detrending methods.

Supplementary Table 1. Descriptions of the five tree-ring sampling sites on the southeastern TP.

Site name	Latitude/Longitude	Elevation	Time span	Species	No. of trees(cores)
**ZG**	29°59′N/97°55′E	3900-4100m	1509-2010	Balfour spruce	69
**BD**	30°05′N/97°25′E	4200-4305m	1512-2011	Balfour spruce	20
**RW**	29°31′N/96°45′E	4280-4337m	1635-2010	Balfour spruce	25
**CD**	31°04′N/96°58′E	4150m	1688-2000	Balfour spruce	19(33)
**ZG2**	29°59′N/97°54′E	4500m	1653-2000	Balfour spruce	20(28)

Note: the site information for ZG and BD was also listed in our previous publication[1], and site information for CD and ZG2 are also available in our previous paper[2].

Supplementary Table 2. Correlation coefficients among the individual TRW site chronologies.

	CD_TRW	RW_TRW	ZG1_TRW	ZG2_TRW
RW_TRW	*r*=0.66, *p*<0.0001 n=301			
ZG1_TRW	*r*=0.60, *p*<0.0001, n=301	*r*=0.63, *p*<0.0001, n=311		
ZG2_TRW	*r*=0.55, *p*<0.0001, n=301	*r*=0.48, *p*<0.0001, n=301	*r*=0.59, *p*<0.0001, n=301	
BD_TRW	*r*=0.47, *p*<0.0001, n=301	*r*=0.51, *p*<0.0001, n=311	*r*=0.65, *p*<0.0001, n=311	*r*=0.57, *p*<0.0001, n=301

Supplementary Table 3. Correlation coefficients among the individual MXD site chronologies.

	CD_MXD	RW_MXD	ZG1_MXD	ZG2_MXD
RW_MXD	*r*=0.49<br>*p*<0.0001<br>n=301			
ZG1_MXD	*r*=0.42<br>*p*<0.0001<br>n=301	*r*=0.28<br>*p*<0.0001<br>n=311		
ZG2_MXD	*r*=0.68<br>*p*<0.0001<br>n=301	*r*=0.49<br>*p*<0.0001<br>n=301	*r*=0.53<br>*p*<0.0001<br>n=301	
BD_MXD	*r*=0.38<br>*p*<0.0001<br>n=301	*r*=0.25<br>*p*<0.0001<br>n=311	*r*=0.67<br>*p*<0.0001<br>n=311	*r*=0.44<br>*p*<0.0001<br>n=301

Supplementary Table 4. **Correlation coefficients between tree-ring residual series (TMres1, 2 and 3) and instrumental temperature residuals (i.e., summer temperature minus winter temperature) in individual station of the study area and the regional average over their common period.**

Meteorological station	Latitude	Longitude	Altitude (m asl)	Period	*r*1	*r*2	*r3*
Nangqian	32°12′N	96°29′E	3644	1958-2011	0.47	0.41	0.45
Batang	30°00′N	99°06′E	2589	1960-2011	0.64	0.61	0.62
Daocheng	29°03′N	100°18′E	3728	1958-2011	0.51	0.50	0.48
Dege	31°48′N	98°35′E	3184	1958-2011	0.55	0.52	0.53
Garze	31°37′N	100°00′E	3394	1952-2011	0.44	0.37	0.43
Litang	30°00′N	100°16′E	3949	1953-2011	0.49	0.40	0.49
Xinlong	30°56′N	100°19′E	3000	1961-2011	0.65	0.62	0.62
Bomi	29°52′N	95°46′E	2736	1962-2011	0.54	0.47	0.52
Zayu	28°39′N	97°28′E	2328	1970-2011	0.52	0.45	0.51
Qamdo	31°09′N	97°10′E	3306	1955-2011	0.65	0.65	0.62
Dengqen	31°25′N	95°36′E	3873	1961-2011	0.50	0.46	0.48
Nyingchi	29°40′N	94°20′E	2992	1961-2011	0.53	0.45	0.52
Zuogang	29°40′N	97°50′E	3780	1979-2011	0.62	0.60	0.59
Regional average				1952-2011	**0.61**	**0.56**	**0.58**

r1: correlation coefficient between TMres1 and summer-minus-winter temperature residuals; *r2*: correlation coefficient between TMres2 and summer-minus-winter temperature residuals; *r3*: correlation coefficient between TMres3 and summer-minus-winter temperature residuals. Note: all the correlation coefficients are significant at the level of 0.01. All correlation coefficients were calculated based on the normalized series.

Supplementary Table 5. Statistics of the calibration and verification for the regression models.

Calibration				Verification			
Season	Period	R^2	F	RE	ST	ST1	PMT
Summer	1952-2011	54.8%	70.1	0.51	42+/18-	44+/15-	4.60
Winter	1952-2011	32.5%	26.5	0.25	40+/19-	40+/20-*	3.75
Summer-Winter	1955-2011	42.3%	32.2	0.30	44+/13-	38+/18-*	1.96

RE = reduction error, ST= sign test, ST1= first-difference sign test, PMT = product mean test. The asterisk means at the significant level of 0.05, and all the other statistics are significant at the level of $p < 0.01$.

Supplementary Table 6. Model information of the simulations (11 models, 29 simulation runs) used in this study.

Model	Experiment	Starting year	End year	No.	Forcing
BCC-CSM1.1	P1000	0850-01-16	2000-12-16	2	Nat
CCSM4	P1000	0850-01-16	1850-12-16	2	A
CSIRO-Mk3L-1-2	P1000	0851-01-16	1850-12-16	1	B
FGOALS-gl	P1000	1000-01-16	1999-12-16	2	C
FGOALS-S2.0	P1000	0850-01-16	1850-12-16	1	D
GISS-E2-R	P1000	0850-01-16	1850-12-16	13	E
HadCM3	P1000	0850-01-16	1850-12-16	1	F
IPSL-CM5A-LR	P1000	0850-01-16	1850-12-16	2	G
MIROC-ESM	P1000	0850-01-16	1849-12-16	2	H
MPI-ESM-P	P1000	0850-01-16	1849-12-16	2	I
MRI-CGCM3	P1000	0850-01-16	1850-12-16	1	N/A

Note that the model simulations of CSIRO-Mk3L-1-2 and HadCM3 are from CMIP3 and the others are from CMIP5.

Expansions of the model name acronyms are available at

http://www.ametsoc.org/PubsAcronymList

The “NO.” means the number of simulations for each model.

A=Sl GHG Vl LU (time-varying over course of simulation), SS Ds SD BC MD OC Oz AA LU (all fixed at or cycled over 1850 values)
B=GHG, Sl, Vl (Sl = orbital parameters and Steinhilber-Beer-Frohlich, Vl = Crowley)
C=GHG, Vl, Sl, SD
D=GHG, SD, Oz, Sl, Vl, SS, Ds,BC, OC
E=GHG LU Sl Vl (including orbital)
F=GHG,Oz,LU,Sl,Vl
G=Nat,Ant,GHG
H=GHG LU Sl Vl (including orbital; GHG includes only CH4 and N2O; CO2 is predicted by model)
I=GHG,SL,VL,LU
The “N/A” means Pre-industrial conditions with all forcings fixed at 1850 levels.

Supplementary references

1. Duan, J. P. & Zhang, Q. B. A 449 year warm season temperature reconstruction in the southeastern Tibetan Plateau and its relation to solar activity. *J Geophys Res-Atmos* **119**, 11578-11592 (2014).
2. Wang, L., Duan, J. P., Chen, J., Huang, L. & Shao, X. M. Temperature reconstruction from tree-ring maximum density of Balfour spruce in eastern Tibet, China. *Int J Climatol* **30**, 972-979 (2010).
